# Retraction: An Artificial Intelligence Fusion Model for Cardiac Emergency Decision Making: Application and Robustness Analysis

**DOI:** 10.2196/98670

**Published:** 2026-04-28

**Authors:** JMIR Publications Editorial Office

**Affiliations:** 1 JMIR Publications Toronto, ON

The JMIR Publications Editorial Office is retracting the article:

An Artificial Intelligence Fusion Model for Cardiac Emergency Decision Making: Application and Robustness Analysis [1]

This action is being taken due to identified concerns regarding potential manipulation of the submission process, authorship, and the relevance of several references.

The concerns with this publication were identified during an investigation of a series of articles with related concerns. The authors were contacted and offered an opportunity to comment on these findings and the proposed retraction but did not respond to any attempts at communication.
